# ERGPNet: lesion segmentation network for COVID-19 chest X-ray images based on embedded residual convolution and global perception

**DOI:** 10.3389/fphys.2023.1296185

**Published:** 2023-11-13

**Authors:** Gongtao Yue, Chen Yang, Zhengyang Zhao, Ziheng An, Yongsheng Yang

**Affiliations:** ^1^ School of Computer Science, Xijing University, Xi’an, China; ^2^ School of Information and Navigation, Air Force Engineering University, Xi’an, China; ^3^ School of Integrated Circuits, Anhui University, Hefei, China

**Keywords:** COVID-19, chest X-ray, lesion area, encoder-decoder, segmentation

## Abstract

The Segmentation of infected areas from COVID-19 chest X-ray (CXR) images is of great significance for the diagnosis and treatment of patients. However, accurately and effectively segmenting infected areas of CXR images is still challenging due to the inherent ambiguity of CXR images and the cross-scale variations in infected regions. To address these issues, this article proposes a ERGPNet based on embedded residuals and global perception, to segment lesion regions in COVID-19 CXR images. First, aiming at the inherent fuzziness of CXR images, an embedded residual convolution structure is proposed to enhance the ability of internal feature extraction. Second, a global information perception module is constructed to guide the network in generating long-distance information flow, alleviating the interferences of cross-scale variations on the algorithm’s discrimination ability. Finally, the network’s sensitivity to target regions is improved, and the interference of noise information is suppressed through the utilization of parallel spatial and serial channel attention modules. The interactions between each module fully establish the mapping relationship between feature representation and information decision-making and improve the accuracy of lesion segmentation. Extensive experiments on three datasets of COVID-19 CXR images, and the results demonstrate that the proposed method outperforms other state-of-the-art segmentation methods of CXR images.

## 1 Introduction

COVID-19 is an acute respiratory infectious disease. The patients usually have uncertain symptoms such as ground-glass opacity, bilateral lower lobe consolidation (Zhang et al., 2023), diffuse airspace disease (Bougourzi et al., 2023), and pleural effusion (Jacobi et al., 2020) in the lungs. Accurately determining the lung disease areas of COVID-19 patients can help clinicians formulate appropriate treatment to prevent further deterioration of the patient. As an important means in the field of computer-aided diagnosis, image segmentation can assign semantic category information to each pixel. Therefore, it is widely used in practical tasks such as disease judgment (Wang et al., 2021), precise treatment (Lyu et al., 2022), and lesion monitoring (Chowdhury et al., 2020).

**FIGURE 1:**
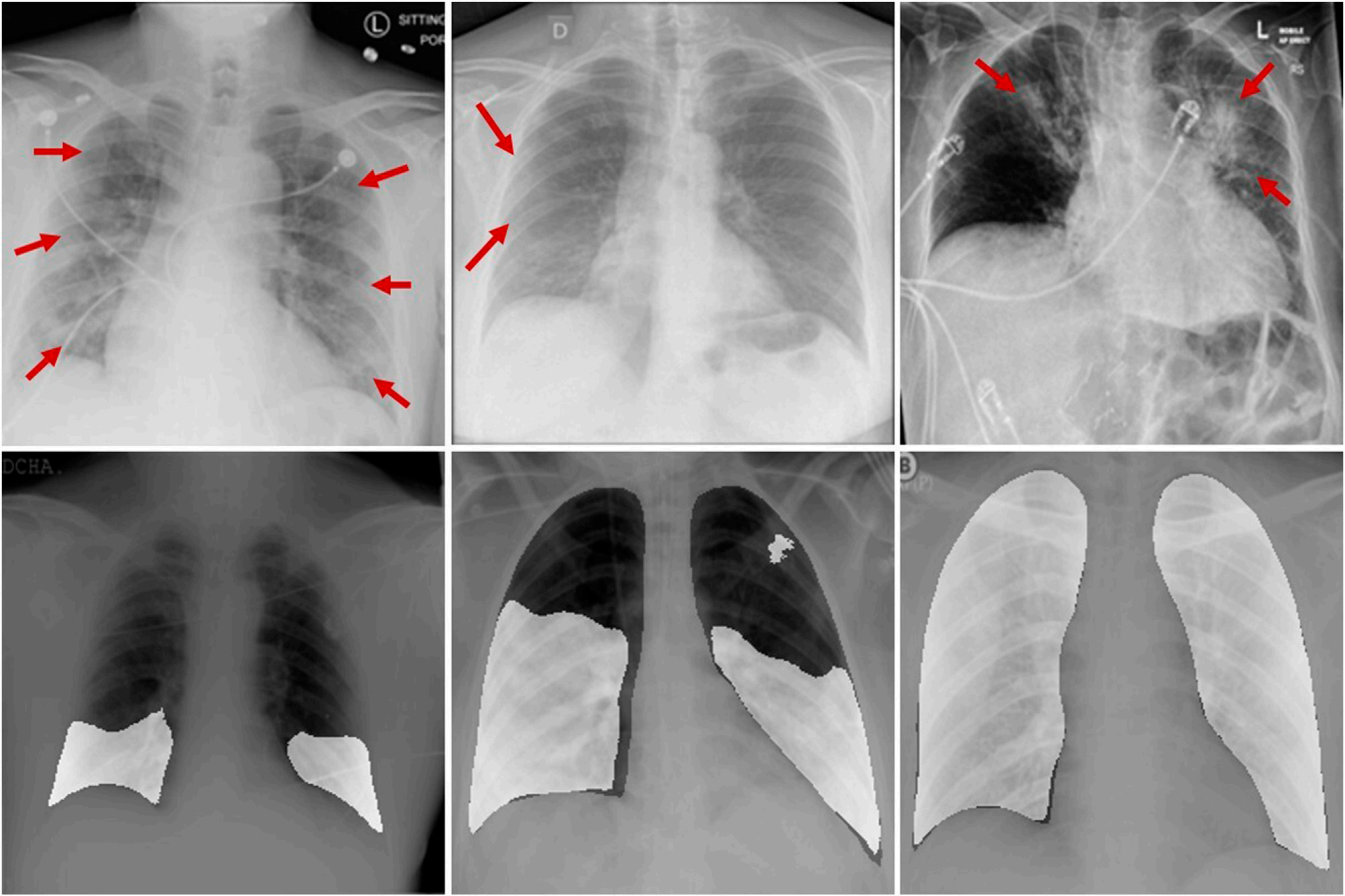
Sample images of infected patients, where the red arrow points to the lesion area in the first row, and the bright white area in the second row represents the lesion area.

During the epidemic, many COVID-19 image segmentation methods based on deep learning were explored. Such as (Huang et al., 2020; Zhou et al., 2020; Paluru et al., 2021) based on convolutional neural networks, (Bhattacharyya et al., 2022), based on conditional generative adversarial networks, (Tiwari and Jain, 2022), based on lightweight capsule networks, (Jia et al., 2023), based on graph reasoning, and (Joshi et al., 2022; Tiwari et al., 2022)combined with transfer learning. These methods have made effective contributions to the diagnosis and treatment of COVID-19 patients. However, due to the limitations of the receptive field of conventional convolution operations, long-distance dependencies of feature information cannot be established. Therefore, it is difficult to make adequate judgments on diseased pixels when facing the following challenges:

The first challenge is that COVID-19 CXR images are characterized by sparse features and blurred backgrounds, making it difficult to form rich semantic representations. As depicted in the top row of Figure 1, the red arrows indicate the infected areas. However, the image does not exhibit clear infection characteristics, which poses a challenge for the network to accurately distinguish and classify infected pixels. To alleviate this issue, some researchers employ multi-task learning to improve the network’s capability of capturing features of infected pixels. For instance, (Zhao et al., 2022), proposed a cascaded segmentation classification network to suppress the interference of background regions during feature extraction by utilizing prior knowledge from the lung segmentation network. They improved the network’s capability to extract features by combining key point extraction with a deep neural network. (Munusamy et al., 2021). developed a novel Fractal CovNet architecture using Fractal blocks and U-Net for the segmentation of chest CT-scan images to localize the lesion region. (Fan et al., 2022). proposed a segmentation network for COVID-19 infected regions. This network incorporates an edge-guided module and a reverse attention module to fully extract the blurred boundary details of the infected area. (Chen et al., 2023). designed an unsupervised method for COVID-19 segmentation, that utilizes a teacher-student network to learn rotation-invariant features for segmentation. However, multi-task learning imposes an additional computational burden on the network, and traditional cascaded convolutions have limited receptive fields and cannot capture deep feature information within the codec layer. Therefore, these methods struggle to adequately identify the details of infected pixels in COVID-19 CXR images.

The second challenge is that the outline and scale of the infected area in COVID-19 CXR images vary greatly, which increases the difficulty for the network to identify cross-regional weakly correlated features. As shown in the second row of Figure 1, the white area represents the region impacted by COVID-19. However, this change in scale and range blurs the details, making it difficult for the network to establish associations between local and global features, leading to misclassification. Therefore, enhancing the global long-distance dependencies and multi-scale feature mapping relations of the network is essential for alleviating the aforementioned problems. For instance, (Mahmud et al., 2021), designed a horizontal expansion module for the multi-level encoder-decoder structure and combined it with pyramidal multi-scale feature fusion to minimize the semantic gap between features of varying scales. (Wang et al., 2020). proposed an anti-noise Dice loss to effectively handle lung lesions of varying sizes and appearances. (Mahmud et al., 2020). proposed a three-layer attention-based segmentation network, combining a three-layer attention mechanism with parallel multi-scale feature optimization to achieve precise segmentation of COVID lesions. (Yu et al., 2022). improved the network’s ability to perceive features in infection regions at different scales by combining a dual-branch encoder structure with spatial attention. (Li et al., 2022). proposed a multi-level attention-based lightweight segmentation network. It helps the network handle changes in scale by incorporating Atrous Pyramid Pooling at the encoding and decoding bottlenecks. However, most of these methods enhance the network’s global perception ability by using multi-scale convolution kernels or by fusing encoder features from different scales. The detailed information on low-dimensional and high-dimensional features cannot be fully utilized, and the long-distance dependencies of high-order features are ignored. Therefore, it cannot effectively deal with the cross-scale variation of the infected area.

To solve the above problems, this paper proposes a new global perception network (ERGPNet) based on embedded residual convolution. The network mainly consists of Embedded residual module (ERM), global perception module (GPM), attention module, and deep supervision module. The ERM replaces the 3 × 3 convolution kernel which increases the convolution depth within the codec layer. The features inside the encoding and decoding layers are fused through the residual connection so that the network can extract multi-scale features inside the encoding and decoding layer. The GPM performs multi-dimensional perceptual integration of high-dimensional semantic information at the bottleneck and guides the decoder to perceive global semantic information in low dimensions. The attention module respectively performs spatial and category weight corrections on feature information to enhance the network’s sensitivity to target information. Finally, the error of the prediction results is optimized through the deep supervision module. ERGPNet achieved the optimal MIoU indicators of 81.66%, 80.79%, and 81.73% in the COVID-QU-Ex, QaTa-COV19, and COVID-19 CXR enhanced datasets, respectively. The main contributions of this article can be summarized as follows.

(1) ERM is designed to extract deeper and wider feature information inside the encoding and decoding layers to reduce the impact of the inherent ambiguity of COVID-19 CXR images on network segmentation.
(2) GPM is proposed to promote the high-dimensional feature information of the codec structure to form global perception capabilities, and then guide the low-dimensional features to establish dependencies between long-distance feature information, thereby reducing the interference caused by cross-scale lesions on feature recognition.
(3) Spatial and channel attention are designed to correct the weights of feature information at different stages to improve the network’s sensitivity to target information.

## 2 Materials and methodology

### 2.1 Data description

In order to validate the effectiveness of the method proposed in this paper, we conducted extensive experiments on two publicly available datasets and one dataset with enhanced images. Among them, the public datasets COVID-QU-Ex (Tahir et al., 2021) and QaTa-COV19 (Degerli et al., 2021) are from researchers at Qatar University and Tampere University. We only used data for which there was a breakdown of COVID-19, and the details of the data are described below.

The COVID-QU-Ex with 33,920 CXRs including 2913 COVID-19 samples with their corresponding ground-truth segmentation masks. The pixel size is 256 × 256, and the depth is 8-bit. These images are divided into a training set of 1864, a validation set of 466, and a test set of 583.

The QaTa-COV19 with 121,378 CXRs including 9258 COVID-19 samples with their corresponding ground-truth segmentation masks. The pixel size is 224 × 224, and the depth is 8-bit. Among them, 5716 images were used as the training set, 1429 images were used as the validation set, and 2113 images were used as the test set.

In the COVID-19 CXR enhanced dataset, we use contrast-limited adaptive histogram equalization and gamma correction techniques to enhance the original image, and then fuse the two enhanced images with the original image to obtain the final dataset. The COVID-19 CXR enhanced dataset contains 2400 images with a pixel size of 256 × 256 and a depth of 24-bit. There are 1600 images as the training set, 400 images as the test set, and 400 images as the validation set.

### 2.2 Overview of the network

**FIGURE 2:**
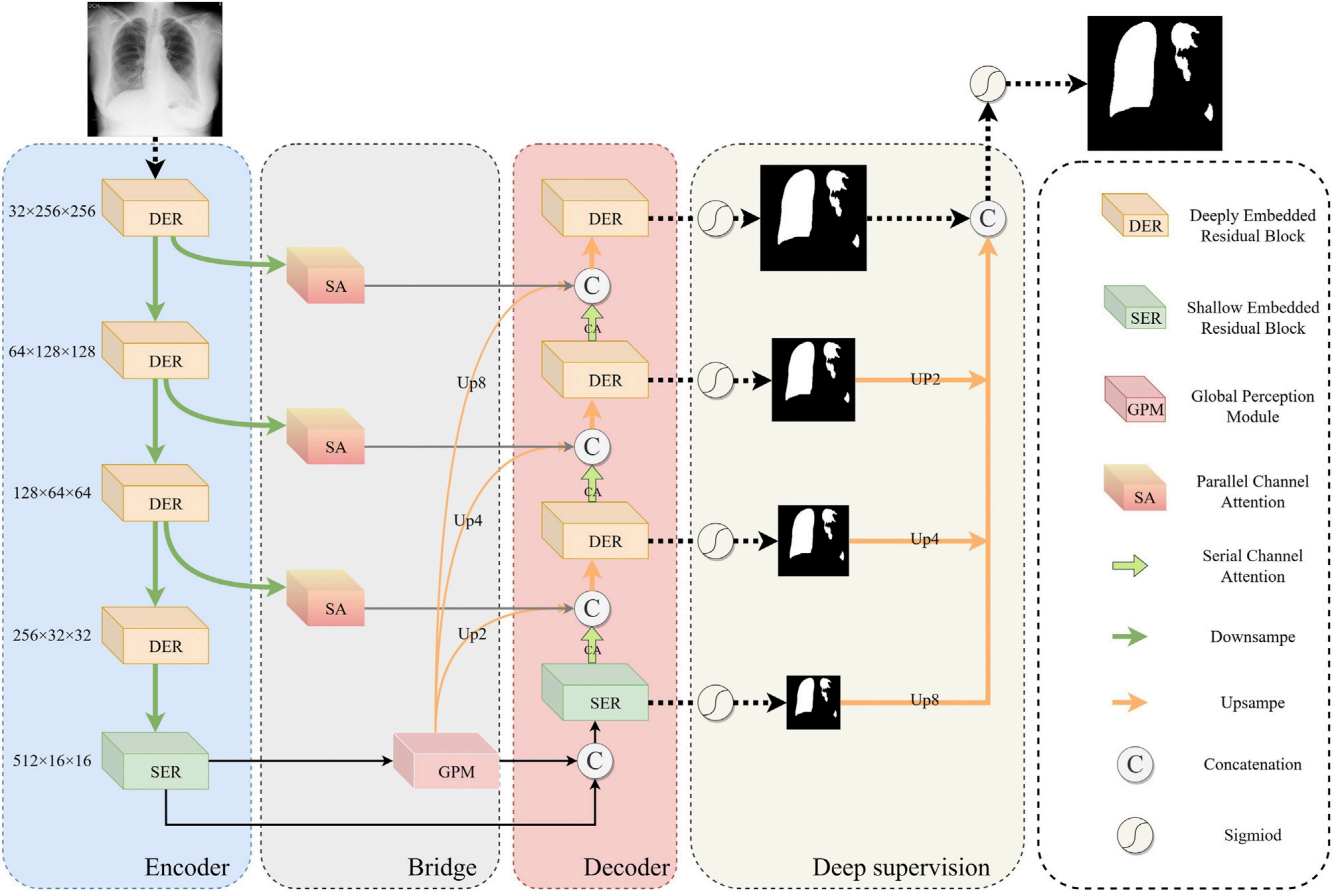
Overall architecture of the ERGPNet.

The overall architecture of ERGPNet is shown in Figure 2, which includes ERM, GPM, attention module, and deep supervision. ERM consists of deep embedded residuals (DER) and shallow embedded residuals (SER), which extract low-dimensional features and high-dimensional features, respectively, and mutually enhance the information obtained from each other. GPM radiates the global perception ability of high-dimensional features to low-dimensional space, guides the fusion of global contextual information and captures feature relationships between cross-scale pixels. The attention module consists of parallel spatial attention and serial channel attention, which enhance the network’s sensitivity to target regions and target channels, respectively, while reducing the influence of noise information on network discrimination. The deep supervision enables the network to calculate the loss in more detail, thereby achieving optimal prediction results.

### 2.3 Embedded residual module

The network needs to utilize more comprehensive information in order to establish an accurate mapping relationship between features. Most convolutional neural networks, however, utilize two linear convolutions of size 3 × 3 in the encoder-decoder layer to extract features. This method limits the receptive field of the network at the encoder-decoder layer and disregards deeper details, leading to the inability to accurately identify infected pixels. Inspired by U2-Net (Qin et al., 2020) and the residual structure, we propose ERM to extract deeper and wider feature information inside the encoder-decoder layer. Specifically, ERM has two structures, including DER and SER.

**FIGURE 3:**
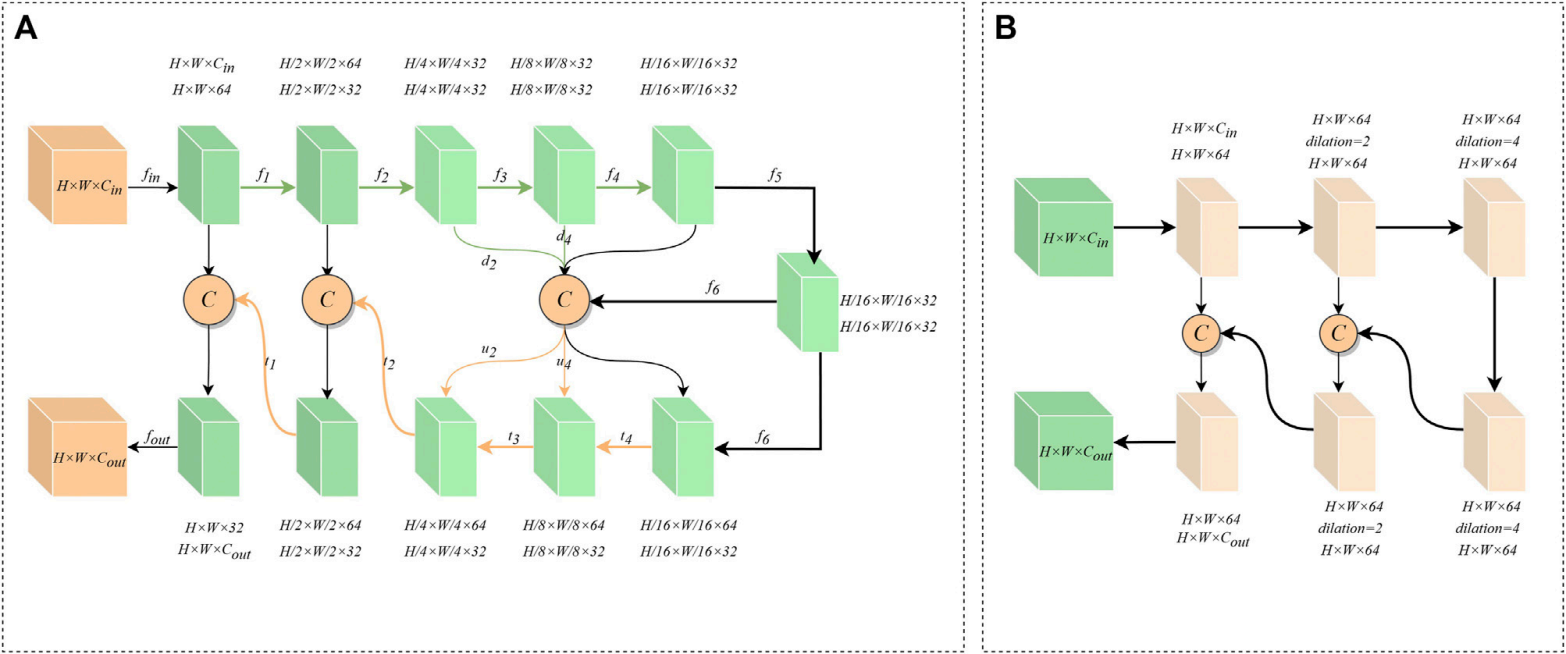
**(A)** Deep Embedding Residual Structure. **(B)** Shallow embedding residual structure.

The structure of DER is shown in Figure 3A. First, the input feature $f_{in}$ is sequentially passed through two convolution blocks to extract shallow features $\left\{f_{i}\left(i=1,2\right)\right\}$ . Then, the shallow semantic features obtained are inputted into four convolution blocks successively to extract deep features of different scales $\left\{f_{i}\left(i=3,4,5,6\right)\right\}$ . Among them, shallow features highlight local fine-grained information, while deep features have abstract information with better generalization. In addition, we utilize the residual connection to merge the shallow features $\left\{f_{i}\left(i=1,2\right)\right\}$ with the features $\left\{t_{i}\left(i=1,2\right)\right\}$ during the feature recovery process. Then, the merged features are inputted into the corresponding feature recovery convolution block, which emphasizes the representation of detailed information. Fusing cross-scale deep features $\left\{f_{i}\left(i=3,4,5,6\right)\right\}$ to obtain $f_{c}$ :

$$f_{c}=Cat\left\{D_{2}\left(f_{3}\right),D_{4}\left(f_{4}\right),f_{5},f_{6}\right\}$$ (1)

Where $Cat\left\{∙\right\}$ represents the channel concatenation operation, $D_{n}$ means downsampling by a factor of n . Then $f_{c}$ is upsampled by different multiples and input into the corresponding feature recovery convolution block to obtain $\left\{t_{i}\left(i=2,3,4\right)\right\}$ respectively. The information from each feature recovery convolution block is fused with multi-scale features $f_{c}$ , resulting in the extraction of richer information. The calculation process is given as follows:

$$t_{4}=ReLU\left\{BN\left\{Conv\left(Cat\left(f_{c},f_{6}\right)\right)\right\}\right\}$$ (2)

$$t_{3}=ReLU\left\{BN\left\{Conv\left(Cat\left({U_{2}(f}_{c}),U_{2}\left(t_{4}\right)\right)\right)\right\}\right\}$$ (3)

$$t_{2}=ReLU\left\{BN\left\{Conv\left(Cat\left({U_{4}(f}_{c}),U_{2}\left(t_{3}\right)\right)\right)\right\}\right\}$$ (4)

$$t_{1}=ReLU\left\{BN\left\{Conv\left(Cat\left(f_{2},U_{2}\left(t_{2}\right)\right),U_{2}\left(t_{2}\right)\right)\right\}\right\}$$ (5)

$$f_{out}=ReLU\left\{BN\left\{Conv\left(Cat\left(f_{1},U_{2}\left(t_{1}\right)\right),U_{2}\left(t_{1}\right)\right)\right\}\right\}$$ (6)

The structure of SER is shown in Figure 3B. Because the feature information of the underlying encoder-decoder block has low resolution and high abstraction characteristics, an excessively deep convolutional structure can lead to overfitting of features. Therefore, SER is designed with only three layers of convolutional extraction blocks. At the same time, we use dilated convolutions with different parameters instead of downsampling to prevent the loss of high-dimensional abstract information. And the cross-level feature information is integrated through the residual connection. Furthermore, the information flow across layers is integrated via residual connections to increase the feature-aware range of convolutional blocks. Thus, SER can effectively capture valuable information in high-dimensional features.

### 2.4 Global perception module

The cross-scale variation of infected regions in COVID-19 CXR images poses a great challenge for network segmentation. Usually, low-dimensional semantic information is helpful in identifying small-scale detail features, while targets with large scale changes often require high-dimensional information with global perception capabilities as a guide. While ASPP (Chen et al., 2017) can capture cross-regional features through multi-scale convolution kernels, FPN (Lin et al., 2017) can obtain long-range feature dependencies by fusing prediction information at different scales. However, these methods may cause the repulsion of features of different scales, resulting in the loss of some feature information. Therefore, after fully considering the characteristics of the encoder-decoder structure network, we have designed a simple and effective GPM at the bottleneck. This module guides the generation of low-dimensional features by leveraging the global awareness of high-dimensional features.\

**FIGURE 4:**
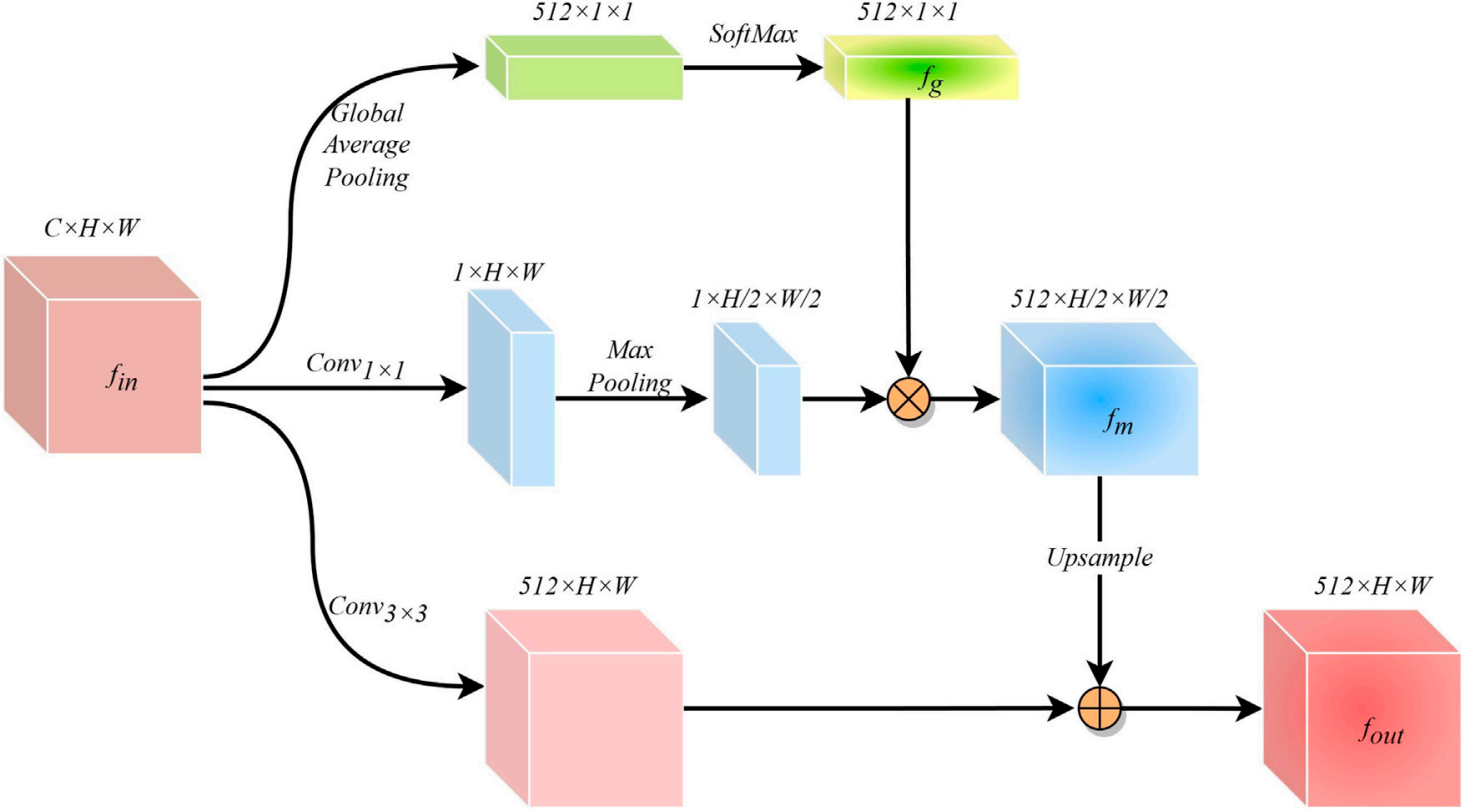
The structure of the global perception module.

The structure of the GPM is shown in Figure 4. The input feature information $f_{in}$ is extracted respectively by three different feature extraction methods. First, global average pooling is used to compress $f_{in}$ in the spatial dimension, reducing the amount of feature calculation while establishing the association between channel feature information and spatial feature information. The calculation process is given as follows:

$$F^{c}=\sum_{y=1}^{16}\left(\sum_{x=1}^{16}f_{xy}^{c}\right)\times \frac{1}{16}\times \frac{1}{16}$$ (7)

$$f_{g}=SoftMax\left\{Cat\left(F^{1},F^{2},F^{3},\cdots ,F^{512}\right)\right\}$$ (8)

Where $C\in \left(1,2,3,\cdots ,512\right)$ represents the channel of the feature, $F^{c}$ represents the feature of channel C after spatial pooling, $f_{xy}^{c}$ represents the eigenvalue with coordinates are $\left(x,y\right)$ on channel C , $Cat\left\{∙\right\}$ represents the channel concatenation operation. Each point on the one-dimensional feature $f_{g}$ after pooling contains feature information of a spatial plane. Secondly, the channel dimension of the feature $f_{in}$ is compressed to one dimension through 1 × 1 convolution, and the relationship between channels is established, so that the features of different channels can be learned interactively. Then, the one-dimensional channel feature map is spatially downsampled by a factor of 2, and the downsampling feature is matrix multiplied by the feature $f_{g}$ to obtain $f_{m}$ :

$$f_{m}=f_{g}⊗\left\{Maxpool\right.\left\{{{Conv}_{1\times 1}\left(F\right.}_{in}\right.)\}\}$$ (9)

Where ⊗ represents matrix multiplication, and ${Conv}_{1\times 1}$ means 1 × 1 convolution operation. Each pixel in $f_{m}$ has channel and spatial weight information. Then, use 3 × 3 convolution to extract the feature of $f_{in}$ , and fuse it with the upsampling by a factor of 2 feature $f_{m}$ to obtain the final output feature $f_{out}$ :

$$f_{out}={Conv}_{3\times 3}\left(f_{in}\right)⊕Upsample\left(f_{m}\right)$$ (10)

Where ⊕ denotes elementwise summation. Each pixel in the output feature $f_{out}$ perceives the information of other pixels. Finally, the feature $f_{out}$ is upsampled and fused to the decoder side to provide guidance for low-dimensional perceptual global information. This helps improve the robustness of the network when extracting features across scales.

### 2.5 Attention mechanism module

The attention mechanism can assign different weights to the feature information in order to enhance the network’s ability to respond to the target area and category. However, since CXR images have more blurred features than natural images, the conventional single attention mechanism cannot maintain high sensitivity to feature information. To enhance the network’s ability to perceive feature information of COVID-19 CXR images, we redesign the attention module. Specifically, in the feature information transfer process of the encoder-decoder structure network, the encoding end is more inclined to extract regional feature information, while the decoding end is more inclined to extract category feature information. Therefore, we designed parallel spatial attention and serial channel attention to improve the sensitivity of the network to regional information and category information, respectively.

**FIGURE 5:**
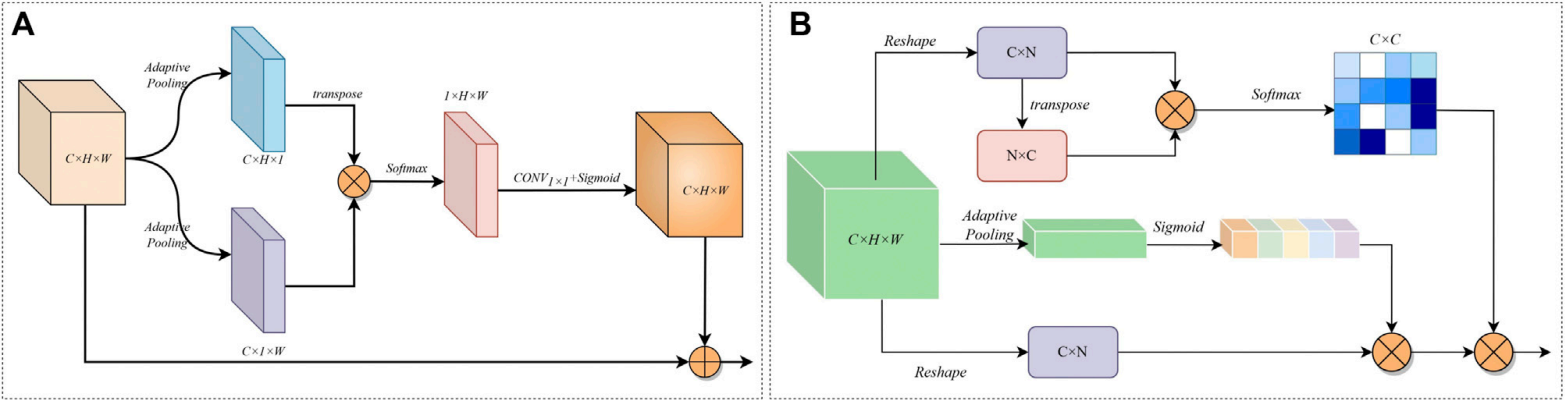
**(A)** Structure of Parallel Spatial Attention Modules. **(B)** Structure of the serial channel attention module.

Figure 5A shows the parallel spatial attention. Given an encoder output feature $f^{e}\in R^{C_{e}\times H_{e}\times W_{e}}$ , where $e\in \left\{1,2,3\right\}$ denote the features output by different encoding layers, C , H , and W denote the depth, height, and width of the feature, respectively. Then, two pooling kernels are used to reshape the feature $f^{e}$ in the height and width dimensions to obtain the two-dimensional feature matrix of the feature map in the height and width dimensions. The calculation process is given as follows:

$$f_{w}^{e}=\frac{1}{w}\sum_{w=1}^{m}f^{e}\left(c,h,w_{m}\right)$$ (11)

$$f_{h}^{e}=\frac{1}{w}\sum_{w=1}^{n}f^{e}\left(c,h_{n},w\right)$$ (12)

Where m and n represents the number of two-dimensional feature matrices in the width and height dimensions, respectively. Then, we transpose the feature matrix $f_{w}^{e}\in R^{C_{e}\times H_{e}}$ to obtain $f_{w}^{e}\in R^{H_{e}\times C_{e}}$ , and subsequently perform matrix multiplication with $f_{h}^{e}\in R^{C_{e}\times W_{e}}$ to obtain $f_{c}^{e}\in R^{H_{e}\times w_{e}}$ :

$$f_{c,mn}^{e}=\frac{\exp\left(f_{c=1,mn}^{e}\right)}{\sum_{1}^{c}\exp\left(f_{c=1,mn}^{e}\right)}$$ (13)

Where $f_{c,mn}^{e}$ represents the feature pixel of point $\left(m,n\right)$ in the feature matrix of $f_{c}^{e}$ . Then, apply SoftMax processing to obtain $f^{e}\in R^{1\times H_{e}\times W_{e}}$ . Next, use a 1×1 convolution operation and Sigmoid activation to obtain the output feature $F^{e}\in R^{C_{e}\times H_{e}\times W_{e}}$ . Finally, it is fused with the input feature $f^{e}$ , and the feature map M corrected by spatial attention is output:

$$M=f^{e}+Sigmoid{\left\{Conv\right.}_{1\times 1}\left(SoftMax\right.\left(f^{e}\right))\}$$ (14)

Compared to the previous method of directly connecting feature information between encoders and decoders, the use of spatial attention correction can enhance the representation of spatial feature information and improve the network’s sensitivity to regional features.

Figure 5B shows the serial channel attention. For the features $f^{d}\in R^{C_{d}\times H_{d}\times W_{d}}$ output by the decoder layer, where $d\in \left\{2,3,4\right\}$ , represent the features output by different solution layers. First, reshape it as $f^{d}\in R^{C_{d}\times N_{d}}$ , where $N_{d}=H_{d}\times W_{d}$ . Then, matrix multiplication is performed on the transposed matrix of $f^{d}$ and $f^{d}$ . After SoftMax processing, the channel feature matrix $f^{d}\in R^{C_{d}\times C_{d}}$ is obtained:

$$f_{ij}^{d}=\frac{\exp\left(f_{ij}^{d}\right)}{\sum_{i=1}^{C_{d}}\exp\left(f_{ij}^{d}\right)}$$ (15)

Where $\left(i,j\right)$ represents the number of different channels of $f^{d}$ , and $f_{ij}^{d}$ represents the influence of channel i on channel j . Perform adaptive pooling and Sigmoid operation on the feature $f^{d}\in R^{C_{d}\times N_{d}}$ and then multiply it with $f^{d}\in R^{C_{d}\times N_{d}}$ to obtain $f_{c}^{d}\in R^{C_{d}\times N_{d}}$ :

$$f_{c}^{d}=f^{d}⊗Sigmoid\left.\left(Adpool{\left(f\right.}^{d}\right)\right)$$ (16)

Finally, perform matrix multiplication with the channel feature matrix $f^{d}\in R^{C_{d}\times C_{d}}$ to obtain the final output matrix K :

$$K={\delta (f}_{c}^{d}⊗f^{d})$$ (17)

Where δ is a learnable parameter initialized from 0 . This method combines two techniques: non-local autocorrelation matrix operation and self-setting pooling. The goal is to enhance the interdependence between channel features and improve the network’s sensitivity to the channel response of the target category.

### 2.6 Deeply supervised loss function

Deep supervision can improve the reliability of the network’s prediction outcomes. Therefore, this paper uses deep supervision to optimize the training process of ERGPNet. Specifically, we fuse feature prediction losses at different depths at the decoder side to guide the network to make feature information decisions. The calculation process is given as follows:

$$L=\sum_{d=1}^{d}W_{p}^{d}L_{p}^{d}+WL_{p}$$ (18)

Where $L_{p}^{d},\left(d=1,2,3,4\right)$ represents the loss of each layer in the encoder prediction map. $W_{p}^{d}$ denotes the weight of each layer in the encoder prediction loss. $L_{p}$ signifies the loss after merging the multi-level prediction map, and W represents the weight used to merge the multi-level prediction loss. For each level of loss L , we use binary cross entropy to calculate it. The calculation process is given as follows:

$$l=-\sum_{\left(x,y\right)}^{\left(H,W\right)}\left[(G_{\left(x,y\right)}^{t})\log P_{\left(x,y\right)}^{t}+\left(1-G_{\left(x,y\right)}^{t})\log(1-P_{\left(x,y\right)}^{t}\right)\right]$$ (19)

Where $\left(x,y\right)$ are the coordinates of the pixel, $\left(H,W\right)$ is the height and width of the image, $G_{\left(x,y\right)}^{t}$ represents the true label of the feature pixel, and $P_{\left(x,y\right)}^{t}$ represents the predicted label of the feature pixel. By stacking the prediction loss of multiple levels of feature maps, the error of network segmentation results is reduced.

## 3 Experimental results and discussion

### 3.1 Evaluation metrics

We quantitatively evaluate the model’s performance at the pixel level using a confusion matrix. First, the pixels in the infected area are marked as positive, and the background pixels are marked as negative. Then count the following elements: the number of pixels correctly predicted as the positive class (TP); the number of pixels correctly predicted as the negative class (TN); the number of pixels incorrectly predicted as the positive class (FP); and the number of pixels incorrectly predicted as negative class Number of pixels (FN). Finally, we evaluated the model’s performance using the following metrics: Accuracy, Precision, Recall, F1-score, and MIoU. The mathematical definitions of these evaluation metrics are as follows:

$$Accuracy=\frac{TP+TN}{TP+TN+FP+FN}$$ (21)

The accuracy here is the ratio of correctly classified pixels among the overall pixels.

$$Precision=\frac{TP}{TP+FP}$$ (22)

The precision rate here refers to the probability that among the samples predicted to be infected pixels are actually infected pixel samples.

$$Recall=\frac{TP}{TP+FN}$$ (23)

The recall rate here refers to the probability of predicting an infected pixel sample among samples that are actually infected pixels.

$$F1=\left(1+\beta^{2}\right)\frac{Precision\times Recall}{\beta^{2}Precision+Recall}$$ (24)

The F1 here is the harmonic mean of precision and recall. It is often used to measure the overall performance of both when high precision and high recall are required.

$$MIoU=\frac{1}{K+1}\sum_{i=0}^{K}\frac{TP}{FN+FP+TP}$$ (25)

The MIoU is used to evaluate the overlapping ratio between the actual segmentation mask and the predicted segmentation mask.

### 3.2 Implementation details

We conduct experiments on a workstation equipped with an Intel Xeon Gold 8350 CPU @ 2.60 GHz and a 12 GB NVIDIA GeForce RTX 3080Ti. The experimental language used was Python 3.8, and all models were executed in PyTorch 1.10. CUDA 11.3. In the training process, in order to balance memory usage and convergence efficiency, we use Adam optimizer and set $\beta_{1}=0.9$ and $\beta_{2}=0.999$ . The initial learning rate is set to 0.0001, and an adaptive learning rate decay strategy is adopted at the same time. After every 10 epochs, if the loss of the validation set does not decrease, the learning rate is reduced to 0.1 times its original value. We set the batch size to 8, applied a weight decay of 0.0005, and implemented early stopping and gradient clipping techniques to prevent overfitting. Finally, the model weights obtained from training are tested on the test set, and the corresponding evaluation metrics are obtained.

### 3.3 Comparison of different networks

In order to validate the effectiveness of the proposed method, we conducted comparative experiments with other state-of-the-art models using different datasets. Including U-Net (Ronneberger et al., 2015), U-Net++ (Zhou et al., 2019), MiniSeg-Net (Qiu et al., 2021), AttentionU-Net (Oktay et al., 2018), CENet (Gu et al., 2019), COPLE-Net (Wang et al., 2020), and Inf-Net (Fan et al., 2020). To ensure fairness, we use the same training parameters and evaluation methods for all networks.

**FIGURE 6:**
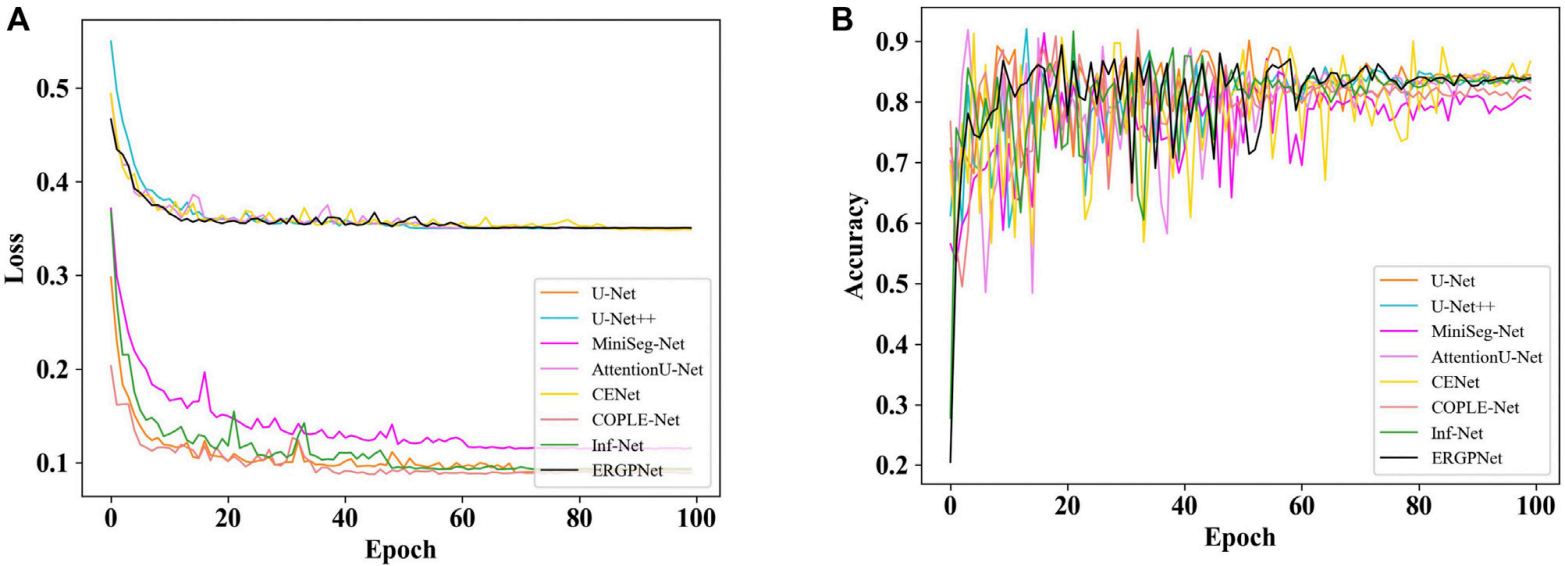
**(A)** Loss curve on validation set. **(B)** Accuracy curve on the validation set.

Figure 6A shows the loss curves of all networks on the verification set data for 100 epochs. For clarity, the loss curve of the proposed ERGPNet is shown in black. It can be seen that the loss of all networks reaches a balance between 60 and 80 epochs and no longer decreases. This indicates that the network has achieved convergence. Among them, ERGPNet, U-Net++, CENet, and AttentionU-Net utilize deep supervision loss during training, resulting in higher than other networks.

Figure 6B shows the accuracy curves of all networks for 100 epochs on the validation set. Similarly, the accuracy curve of the ERGPNet proposed in this paper is represented in black. It can be observed from the figure that the accuracy curves of the validation set for all networks fluctuate significantly. This fluctuation may be attributed to the complex characteristics of the task of segmenting the infection region in COVID-19 CXR images. Although the fitting process exhibits strong fluctuations, these fluctuations decrease as the Epoch increases, eventually reaching a stable state. And it can be seen that the accuracy of the proposed ERGPNet is better than other methods.

In order to understand the structural advantages of ERGPNet, we compared it with the structures of other networks. The details are as follows.

**FIGURE 7:**
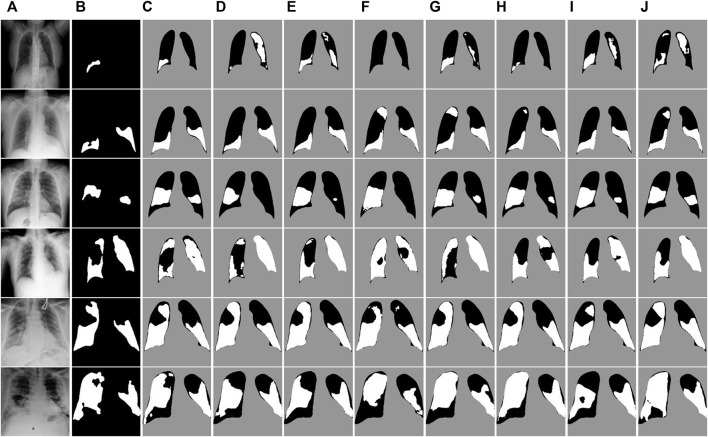
Visual segmentation results on the COVID-QU-Ex test dataset. **(A)** Images. **(B)**Mask. **(C)** Ours. **(D)** U-Net. **(E)** U-Net++. **(F)** MiniSeg-Net. **(G)** Attention-Net. **(H)** CENet. **(I)** COPLE-Net. **(J)** Inf-Net.

**FIGURE 8:**
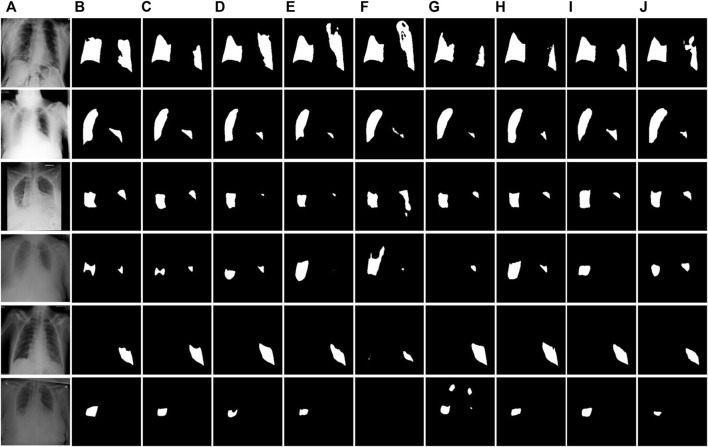
Visual segmentation results of the QaTa-COV19 test dataset. **(A)** Images. **(B)**Mask. **(C)** Ours. **(D)** U-Net. **(E)** U-Net++. **(F)** MiniSeg-Net. **(G)** Attention-Net. **(H)** CENet. **(I)** COPLE-Net. **(J)** Inf-Net.

(1) U-Net: The symmetric up-and-down sampling process and skip connections in this network provide a benchmark for the codec structure. However, due to the single convolution process of U-Net and the simple skip connections between encoders and decoders, network training is prone to overfitting. Therefore, as shown in Tables 1–3, U-Net obtained lower MIoU indicators of 80.47%, 79.56%, and 80.52% in the three data sets, respectively.
(2) U-Net++: This network changes the skip connection method in U-Net and adopts a dense connection method so that the decoder side obtains more information flow. And error correction is performed through in-depth supervision, which further improves the network’s decision-making ability on feature information. However, dense links also cause additional calculations, and no attention is paid to the extraction of multi-scale information. Therefore, Unet++ still cannot have good performance on the COVID-19 segmentation task.
(3) MiniSeg-Net: In order to reduce the computational load of densely connected networks, MiniSeg-Net uses the Downsampler Block and Attentive Hierarchical Spatial Pyramid Module as the basic modules. First, the network feature information is dimensionally reduced, and then the information of different sizes of receptive fields is obtained through multi-scale feature fusion. This network has minimal experimental parameters and training speed but cannot obtain enough rich feature information. Therefore, there are many missed detections in the determination of infected pixels. See Figure 7, 8, 9.
(4) AttentionU-Net: This network adds attention-gating units in the skip connection process, mainly to highlight the salient features of specific local areas. However, single attention cannot enhance the network’s sensitivity to target category information, so there will be some errors in determining the category, resulting in mediocre performance.
(5) CE-Net: Since continuous pooling will lead to the loss of spatial information, a contextual feature extraction module is proposed in CE-Net to capture broader and deeper semantic features by cascading multi-scale atrous convolutions. And further obtain contextual information through multi-scale pooling operations. Because this network has powerful multi-scale spatial information extraction capabilities, it has good performance on the COVID-19 segmentation task. The MIoU in Table 1 and Table 3 reached sub-optimal indicators of 81.39% and 81.51%, respectively.
(6) COPLE-Net: An anti-noise framework is proposed in this network, which adaptively integrates the student model and the teacher model to suppress the influence of noise. And capture multi-scale feature information through residual connections and the ASPP module. However, because the network uses a bridge connection of simple compression channels, it is easy to create a semantic gap, which affects the performance of the network.
(7) Inf-Net: This network extracts edge information from low-dimensional features through the explicit edge attention module, and then aggregates high-level features through parallel partial decoders to generate regional information. Finally, the reverse attention module is used to guide the connection between edge information and regional information. This method corrects the network’s attention to the target area but ignores the connection of hidden layer features outside the domain. This causes the network to over-segment long-distance areas, as shown in Figure 8.

**TABLE 1:** Quantitative evaluation metrics on the COVID-QU-Ex dataset, the optimal and suboptimal indicators are marked with bold values.

Methods	Accuracy	Precision	Recall	F1-score	MIoU
**U-Net** Ronneberger et al. (2015)	0.9593	0.8775	0.8993	0.8821	0.8047
**U-Net++** Zhou et al. (2019)	0.9598	0.8914	0.8934	0.8871	0.8124
**MiniSeg-Net** Qiu et al. (2021)	0.9500	0.8617	0.8742	0.8617	0.7750
**AttentionU-Net** Oktay et al. (2018)	0.9600	0.8933	0.8939	**0.8884**	0.8137
**CENet** Gu et al. (2019)	0.9606	**0.8942**	0.8935	**0.8884**	**0.8139**
**COPLE-Net** Wang et al. (2020)	**0.9609**	0.8826	0.9010	0.8859	0.8105
**Inf-Net** Fan et al. (2020)	0.9606	0.8842	**0.9043**	0.8877	0.8128
**Ours**	**0.9628**	**0.8972**	**0.9055**	**0.8927**	**0.8166**

**TABLE 2:** Quantitative evaluation metrics on the QaTa-COV19 dataset, the optimal and suboptimal indicators are marked with bold values.

Methods	Accuracy	Precision	Recall	F1-score	MIoU
**U-Net** Ronneberger et al. (2015)	0.9634	0.8600	0.9020	0.8734	0.7956
**U-Net++** Zhou et al. (2019)	0.9621	0.8515	0.9035	0.8700	0.7894
**MiniSeg-Net** Qiu et al. (2021)	0.9499	0.8340	0.8608	0.8399	0.7496
**AttentionU-Net** Oktay et al. (2018)	0.9630	0.8532	**0.9037**	0.8702	0.7908
**CENet** Gu et al. (2019)	0.9647	**0.8642**	0.9002	**0.8747**	0.7989
**COPLE-Net** Wang et al. (2020)	0.9610	0.8336	0.8649	0.8490	0.8011
**Inf-Net** Fan et al. (2020)	**0.9660**	**0.8715**	0.9013	0.8730	**0.8045**
**Ours**	**0.9654**	0.8541	**0.9055**	**0.8793**	**0.8079**

**TABLE 3:** Quantitative evaluation on the feature-augmented COIVD-19 image dataset, the optimal and suboptimal indicators are marked with bold values.

Methods	Accuracy	Precision	Recall	F1-score	MIoU
**U-Net** Ronneberger et al. (2015)	0.9610	0.8782	0.9008	0.8843	0.8052
**U-Net++** Zhou et al. (2019)	**0.9623**	0.8941	0.8974	0.8871	0.8125
**MiniSeg-Net** Qiu et al. (2021)	0.9517	0.8712	0.8755	0.8628	0.7821
**AttentionU-Net** Oktay et al. (2018)	0.9605	0.8925	0.8949	0.8867	0.8146
**CENet** Gu et al. (2019)	**0.9631**	**0.8958**	0.8975	**0.8891**	**0.8151**
**COPLE-Net** Wang et al. (2020)	0.9614	0.8827	0.9046	0.8848	0.8127
**Inf-Net** Fan et al. (2020)	0.9627	0.8844	**0.9047**	0.8885	0.8134
**Ours**	0.9620	**0.8979**	**0.9065**	**0.8941**	**0.8173**

**FIGURE 9:**
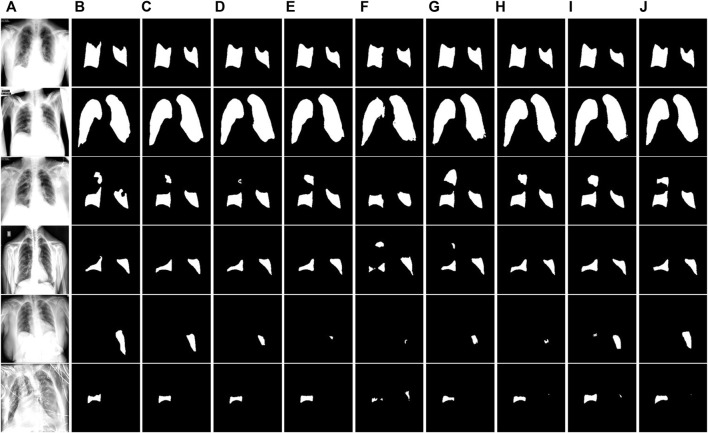
Visual segmentation results on the COVID-enhanced image test set. **(A)** Images. **(B)**Mask. **(C)** Ours. **(D)** U-Net. **(E)** U-Net++. **(F)** MiniSeg-Net. **(G)** Attention-Net. **(H)** CENet. **(I)** COPLE-Net. **(J)** Inf-Net.

Different from the above network structure, ERGPNet changes the feature extraction method within the encoding and decoding layer, can extract multi-scale information within the encoding and decoding layer, and reduces the problem of sparse features caused by the inherent blurriness of COVID-19 CXR images. Different from the skip connection methods of UNet and COPLE-Net, ERGPNet uses spatial attention for optimization in the connection process, which increases the weight of target area information while reducing the semantic gap between codecs. At the same time, the channel attention correction performed in the decoder part enhances the network’s sensitivity to target category information, making the information determination more accurate than other networks. And unlike other networks that extract global information through multi-scale convolution kernels or multi-scale feature fusion, this paper globalizes the high-level semantic information at the bottleneck of the codec structure in different dimensions and establishes the correlation between local features and global features. Therefore, ERGPNet achieved the optimal MIoU of 81.66%, 80.79%, and 81.73% on the three data sets, respectively.

To gain a more detailed understanding of the segmentation performance of the networks, we visually compare the segmentation results of all networks on the test dataset. Figure 7 shows the segmentation results of the COVID-QU-Ex test dataset. The irrelevant background area pixels are indicated in gray, the lung area pixels are indicated in black, and the COVID-19 infected area is indicated in white. It can be observed that our method achieves better detail segmentation results on the small-area infected images in the first to third rows. Additionally, the segmentation error rate of infected pixels is lower compared to other networks. This is because ERB and SER can enable the network to accurately extract features at different levels, achieving a balance and interaction between feature information, and obtaining more comprehensive feature representations. On the large-area infected image segmentation results of the fourth and fifth row images, our method also achieved good performance. This also verifies the robustness of ERGPNet when dealing with infected images of various sizes.

Figure 8 and Figure 9 show the segmentation results of the network on the QaTa-COV19 and COVID-19 enhanced datasets, respectively. Pixels in the infected area are marked as white, while other background pixels are marked as black. As shown, it can be seen that the proposed method is superior, and it allows for more accurate identification of subtle regions, such as lines 3-6 in Figure 8 and lines 4-6 in Figure 9. This is because the ERM, combined with the attention mechanism, enhances the network’s sensitivity to the detailed features of the target area, thereby preventing the loss of information during the segmentation of small areas. And by comparing the infection segmentation results of different scales and contours in Figure 7, Figure 8, and Figure 9, it can be observed that our method is more effective in distinguishing infected areas across various scales. This is because GPM establishes the cross-region dependency of pixel features, which improves the robustness of the network for cross-scale lesion segmentation. Through visual comparison of network segmentation results, we further prove the effectiveness of each module of ERGPNet.

### 3.4 Ablation analysis

In order to assess the effectiveness of ERM, GPM, and attention module in ERGPNet, we conducted the ablation analysis in this section. The hyperparameters are set the same during the experiment to ensure the fairness of the results. Quantitative experimental results are shown in Table 4, where the baseline model represents the simplest U-Net network. Since our proposed ERM can better extract the feature information in the codec layer than the conventional convolution, F1 and MIoU are increased by 0.51% and 0.44%, respectively. GPM enhances the network’s ability to perceive global information, so F1-Score and MIoU increase by 0.27% and 0.22%, respectively. The attention module enhances the sensitivity of the network to target region and channel features, so both F1-Score and MIoU are increased by 0.34%. In summary, each module can increase the segmentation performance of the network to a certain extent.

**TABLE 4:** Ablation studies of ERM, GPM, and MixAttention on the COVID-QU-Ex dataset.

Module	FI-score	MIoU
**Baseline**	0.8821	0.8047
**Baseline + ERM**	0.8872	0.8091
**Baseline + GPM**	0.8848	0.8069
**Baseline + Attention**	0.8855	0.8081
**ERGPNet**	0.8927	0.8166

**FIGURE 10:**
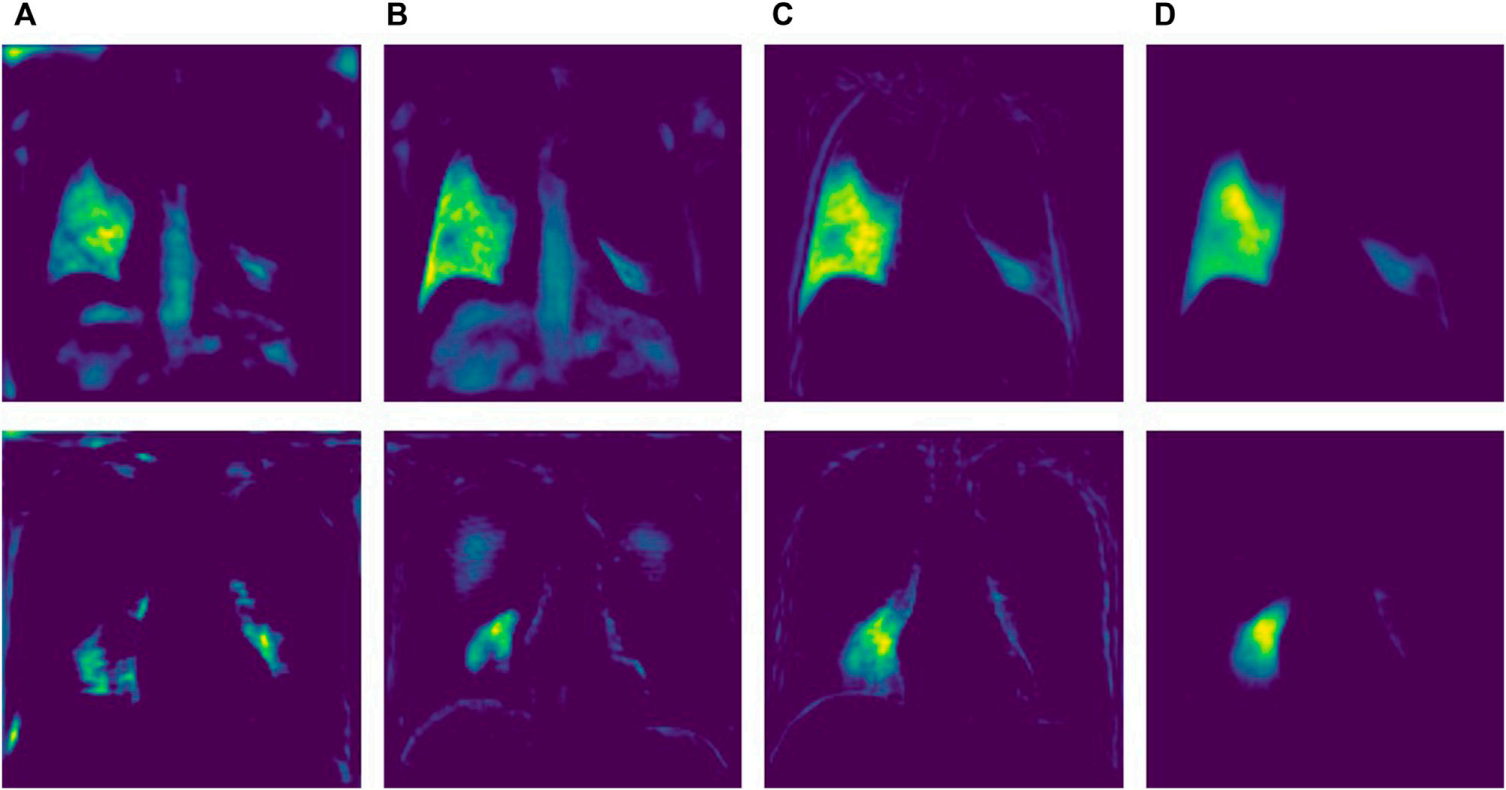
Node characteristics under different modules. **(A)** Baseline Structure **(B)** ERB **(C)** GPM **(D)** Attention.

In order to further verify the performance of each module within the network, we selected a node in the decoder and performed a visual analysis of the node feature map after adding each module. As shown in Figure 10. The feature map in column A is the node features of the ordinary structure, column B is the node features added to ERB, column C is the node features added to GPM, and column D is the node features added to the attention module. First, by comparing columns A and B, we can find that the added ERB module obviously captures richer features. Secondly, it can be seen from column C that the added GPM module makes the network pay attention to the global contour information. Finally, the addition of the attention module obviously enables the network to better focus on the target area and reduces the representation of irrelevant feature information. Overall, each module plays a positive role in the network’s ability to extract feature information.

### 3.5 Feature comparative analysis

**FIGURE 11:**
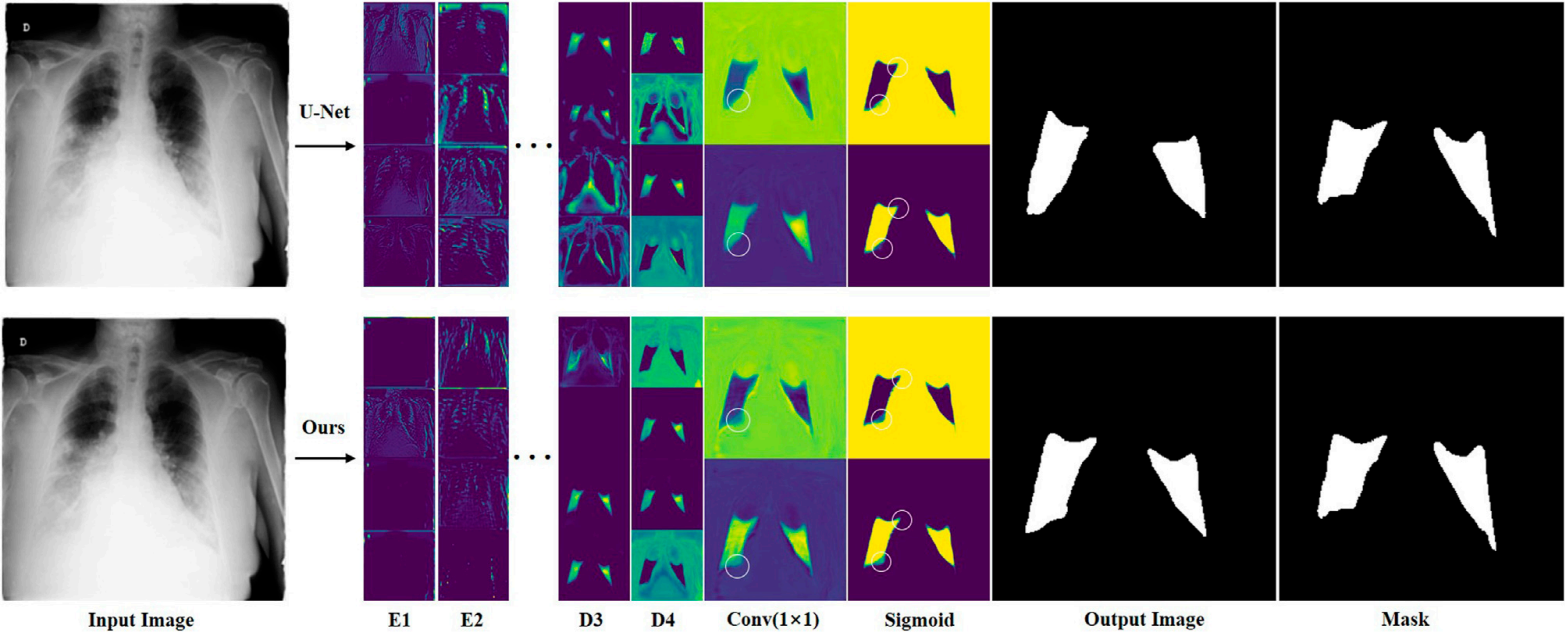
Visual comparison of feature changes between ERGPNet and U-Net.

In order to analyze in detail how features change during network computation, we visually compared the feature maps of ERGPNet and U-Net. Figure 11 shows the output features of the first two layers of encoders, the last two layers of decoders, the 1 × 1 convolutional layer, and the Sigmoid function of the network. We randomly visualized the four feature channels of the codec layer for comparison. Although the features are fuzzy and abstract, it can still be seen that the proposed method has advantages. Since the feature maps output by the 1 × 1 convolutional layer and the Sigmoid function only have background and foreground channels, it is evident that the contours segmented by our method are more detailed and accurate, as shown by the white circles in the Figure 15. This is due to the fact that each of our functional modules is specially designed to improve the network’s feature awareness of infected areas.

### 3.6 Grad-GAM analysis

**FIGURE 12:**
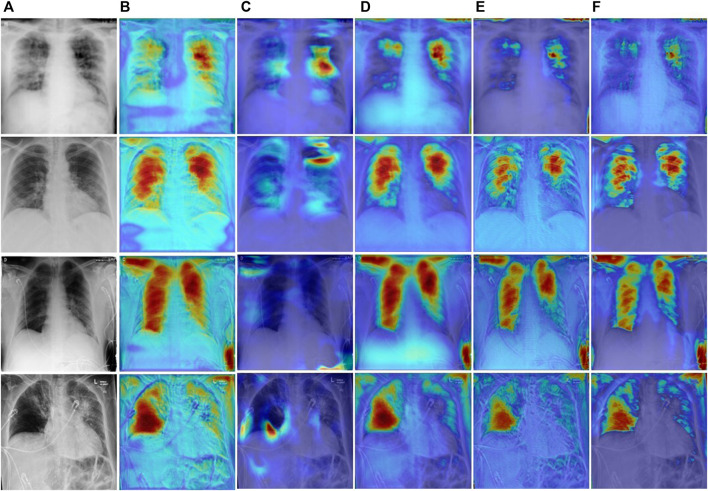
Visual comparison of different network heatmaps. **(A)** Images. **(B)** U-Net++. **(C)**MiniSeg-Net. **(D)** CENet. **(E)** Inf-Net. **(F)** Ours.

To explore the regions of interest during network learning, we use Grad-CAM (Selvaraju et al., 2017) to visualize feature information as heat maps. As shown in Figure 12. The U-Net++ network pays attention to the feature information of many non-target areas, which may be caused by overfitting during feature extraction. Due to the lightweight design of MiniSeg-Net, it is difficult to generate sufficient attention to the target area. In contrast, CE-Net, Inf-Net, and our network can generate sufficient attention to the target region information. But overall, our network is significantly clearer when focusing on infected areas, which also illustrates the robustness and specificity of our network in focusing on COVID-19 features.

## 4 Conclusion

This study proposes a novel ERGPNet network that can accurately segment lesion areas of COVID CXR images with inherent blur and cross-scale lesions. First, we propose an ERB to replace the conventional convolution, which can extract richer information in the encoder-decoder layer. Secondly, GPM is designed to enhance the mapping relationship of global features and reduce the impact of cross-scale changes of infected regions on network segmentation performance. Then, considering the characteristics of the encoder-decoder network, parallel spatial attention and serial channel attention are designed to enhance the network’s sensitivity to pixels in infected regions. Finally, the deep supervision method is used to ensure that the network achieves optimal convergence results. The effectiveness and superiority of the proposed algorithm have been verified through segmentation experiments conducted on three datasets. In addition, ablation experiments and visual analysis also demonstrate the effectiveness of each functional module within the network. However, segmenting infected regions with complex contours is still a challenge, as shown in Figure 9, line 6. Therefore, further improving the network’s ability to identify edge information in infected areas is our future research direction.

## Data Availability

The original contributions presented in the study are included in the article/Supplementary material, further inquiries can be directed to the corresponding author.
